# The Role of L-Cysteine/H_2_S Pathway in CaSRs-Mediated Relaxations in Mouse Bladder Tissue

**DOI:** 10.33549/physiolres.935579

**Published:** 2025-06-01

**Authors:** Fatma AYDINOGLU, Kubra GONBE, Nuran OGULENER

**Affiliations:** 1 Department of Pharmacology, Pharmacy Faculty, Cukurova University, Adana, Republic of Türkiye; 2 Department of Pharmacology, Medical Faculty, Cukurova University, Adana, Republic of Türkiye

**Keywords:** Bladder, CaSRs, Calhex-231, Hydrogen sulfide, L-cysteine, Mouse

## Abstract

The activation of Calcium-Sensing Receptors (CaSRs) reduces detrusor activity in bladder tissues. Also, hydrogen sulfide (H_2_S) produces in bladder tissue and regulates the bladder smooth muscles tone. However, there is no evidence of the interaction between CaSRs and H_2_S in bladder tissue. The aim of this study is to investigate the possible contribution of L-cysteine/H_2_S pathway in CaSRs-mediated relaxation responses in isolated mouse bladder tissue. CaCl_2_ (1, 2, 3, 5, 10 mM) was applied to isolated mouse bladder tissues pre-contracted with carbachol (1 μM). CaCl_2_-induced relaxations were performed in the presence of PAG (10 mM), AOAA (1 mM), and Calhex-231 (5 μM), cystathionine-gamma-lyase (CSE), cystathionine-beta-synthase (CBS) and CaSR inhibitor, respectively. L-cysteine (1 μM-10 mM), an H_2_S substrate, was used to induced a concentration-dependent relaxant response in isolated bladder tissues pre-contracted with carbachol. L-cysteine induced relaxations were performed in the presence of PAG (CSE inhibitor, 10 mM), AOAA (CBS inhibitor, 1 mM) and Calhex-231 (CaSR inhibitor, 5 μM). CaCl_2_-induced relaxations were decreased by PAG and AOAA. Also, Calhex-231 decreased the CaCl_2_-induced relaxant responses. L-cysteine-induced relaxant responses were reduced in the presence of PAG (10 mM) and AOAA (1 mM). Calhex-231 (5 μM) caused a significant decrease in L-cysteine-induced relaxations. Also, Calhex-231 reduced the increase in H_2_S production in the presence of L-cysteine. In addition, CaCl_2_ increased basal H_2_S generation, and PAG (10 mM), AOAA (1 mM) and Calhex-231 (5 μM) reduced the increase in H_2_S production stimulated with CaCl_2_. In conclusion, CSE and CBS-derived endogenous H_2_S formation may, at least in part, contribute to CaSR-mediated relaxation responses, and CaSRs involve in endogenous H_2_S relaxation responses in isolated mouse bladder tissue.

## Introduction

Calcium-sensing receptors (CaSRs) are found in many tissues and induce numerous functions [1]. CaSRs were firstly expressed from the bovine parathyroid gland, where they regulate parathyroid hormone-dependent extracellular calcium homeostasis [2]. CaSRs are G protein-coupled receptors (GPCRs) located on the cell membrane and sense the levels of extracellular Ca^2+^, and activate signaling pathways that modulate calcium homeostasis. The activation of CaSRs by increased extracellular Ca^2+^ or with other CaSRs agonists triggers the phosphatidylinositol-specific phospholipase C (PLC), initiating the formation of diacylglycerol (DAG) and inositol 1,4,5-trisphoshpate (IP_3_), inhibition of adenylyl cyclase and activation of MAPK/ERK1/2 pathway [3,4].

Recent studies have demonstrated that tissues previously considered non-calciotropic, such as vascular smooth muscle may play a role in modulating Ca^2+^ homeostasis [5,6]. Also, it has been shown that CaSRs produced hyperpolarization and vasodilation in mesenteric artery tissues of rat and rabbit [7,8]. CaSRs is also expressed in vascular endothelium [9] and in smooth muscle cells [10,11]. Alam *et al*. suggested that CaSRs-mediated relaxation may contribute to multiple components within the mesenteric vasculature [12]. Bukoski and co-workers have reported that increasing extracellular [Ca^2+^] within the physiological range (from 1 to 5 mM) induces relaxation in rat mesenteric arteries and also showed that the Ca^2+^-induced relaxation was independent of a functional endothelium [13]. Moreover, Ca^2+^-induced relaxation was also attenuated by iberiotoxin and capsaicin, suggesting that Ca^2+^ activates CaSRs on perivascular nerves, leading to the release of a diffusible substance which in turn activates large conductance calcium-activated potassium (BKCa) in vascular smooth muscle cells (VSMCs) [14].

In addition, Calhex-231 (a negative allosteric modulator of CaSR) antagonized the effects of CaCl_2_ and calindol which are positive allosteric modulators of CaSRs [15]. Also, it has been shown that stimulation of endothelial CaSRs induces nitric oxide (NO)- and endothelium derived hyperpolarizing factor (EDHF)-mediated vasorelaxation in pre-contracted arteries. Furthermore, the increase in extracellular Ca^2+^ concentration or calcimimetics stimulates endothelial CaSRs and produces NO, which causes vasorelaxation *via* stimulation of BKCa channels in VSMCs [16–20]. Hydrogen sulfide (H_2_S) was known as a toxic gas in the past. For the first time, H_2_S was synthesized in brain tissue of mammals, and recognized a gaseous neurotransmitter such as NO and carbon monoxide [21]. H_2_S is synthesized through cystathionine gamma lyase (CSE), cystathionine beta synthase (CBS) and 3-mercaptopyruvate sulfurtransferase (3-MST) enzymes in various tissues [21–23]. H_2_S synthesis occurs in the bladder tissue and urothelium of various species, such as mice, rats, pigs, and human, where it plays a role in the regulation of muscle tone and is associated with conditions including overactive bladder [24–29]. Eto and Kimura have been shown that CBS are involved in the regulation of its activity in the presence of Ca^2+^ and calmodulin in brain tissue [30].

Also, recent studies suggest that the elevation in intracellular Ca^2+^ increases CSE activity and H_2_S generation in vascular smooth muscle cells [31,32]. In addition, it has been reported that CSE-induced H_2_S synthesis is enhanced by the activation of CaSRs with CaCl_2_ in vascular tissues [33]. Furthermore, Wang *et al*. found that the CaSRs-induced upregulation of CSE expression and the production of endogenous H_2_S are related to the PLC-IP3 receptor and calcium-calmodulin (CaM) signaling pathways [34]. However, it is not known that CaSRs regulates detrusor activity by which mechanism. Wu *et al*. reported that CaSRs are expressed in the rat bladder urothelium and the activation of these receptors reduces detrusor activity [35]. To our knowledge, there are no studies investigating the possible interaction between CaSRs and H_2_S pathway in bladder tissue. For the first time, we investigated the role of the L-cysteine/H_2_S pathway in CaSRs-mediated responses in mouse bladder tissue. Our data first demonstrate that there is an interaction between L-cysteine/H_2_S pathway and CaSRs, and, CSE/CBS-induced endogenous H_2_S may partly contribute to the relaxation responses due to CaSRs activation in mouse bladder.

## Materials and Methods

### Animals

Swiss albino male mice were used in the experiments. All experimental protocols were approved by the Cukurova University Local Ethics Committee of Animal Experiments (the approval number 3/11/04.05.2023). The animals were kept under a 12 h light/dark cycle and allowed free access to food and water. The present study was followed by the Guide for the Care and Use of Laboratory Animals published by the US National Institutes of Health (Bethesda, MA, USA; NIH Publication No. 85-23 revised 1996).

### Tissue preparation

Male Swiss albino mice, weighing 20–25 g, were used for these experiments. They were killed by stunning and cervical dislocation. The bladder tissue was carefully removed. Strips (0.5 mm wide and 4–5 mm long) from the midportion of the urinary bladder with urothelium were mounted in a (5 ml) organ bath filled with Krebs solution (in mM: NaCl 118.1, KCl 4.7, CaCl_2_ 2.5, MgCl_2_ 6H_2_O 1.2, KH_2_PO_4_ 1.2, NaHCO_3_ 25, glucose 11.5). The bath medium was maintained at 37 °C and gassed with a mixture of 95 % O_2_ and 5 % CO_2_ at pH 7.4. Muscle strips were allowed to equilibrate for 60 min, during which the medium was changed every 15 min. Changes in muscle length were recorded isometrically *via* an isometric transducer (MP35).

### Experimental protocol

In this study, the interaction between CaSRs and the H_2_S pathway was investigated. In the first experiments, the contribution of endogenous H_2_S to the CaCl_2_-induced relaxations was investigated. Firstly, tissues were contracted with carbachol (1 μM) to assess the viability of bladder strips. After the contractions to carbachol were obtained, tissues were washed and incubated for 30 min in Krebs solutions. Then, the tissues were re-contracted with carbachol (1 μM), and after the contractile responses were reached a plateau, bolus CaCl_2_ (1, 2, 3, 5 and 10 mM) was applied for relaxation responses. To demonstrate the role of CaSRs in the CaCl_2_-induced relaxations, experiments were run in the presence of Calhex-231, a CaSRs – specific inhibitor. For this purpose, after the contractile responses to carbachol were obtained, tissues were washed and incubated with 5 μM Calhex-231 for 30 min and then responses to CaCl_2_ were obtained in the same manner. Also, the contribution of endogenous H_2_S to the relaxant responses to CaCl_2_ (1, 2, 3, 5 and 10 mM) was investigated in the presence of propargylglycine (PAG: CSE inhibitor; 10 mM) or aminooxyacetic acid (AOAA: CBS inhibitor; 1 mM). For this purpose, the tissues were washed and incubated with PAG (10 mM) or AOAA (1 mM) for 60 and 30 min, respectively, and then responses to CaCl_2_ were obtained in the same manner. Furthermore, the contribution of CaSRs to the relaxant responses to L-cysteine (H_2_S substrate) was investigated. Firstly, the bladder tissues were contracted with carbachol (1 μM) and relaxation responses were obtained by applying cumulatively L-cysteine (1 μM-10 mM) to the tissues. After the first series of relaxation responses to L-cysteine were obtained, tissues were incubated for 30 min with Krebs solutions and the second series of relaxations were recorded in the same manner. In mouse bladder tissue, to confirm that L-cysteine-induced relaxations are dependent on the endogenous H_2_S, the effects of CSE and CBS enzyme inhibitors on these relaxations were investigated. For this purpose, after the first series of L-cysteine relaxations, the tissues were incubated with PAG (10 mM) or AOAA (1 mM) for 60 and 30 min, respectively, and then the second series of responses to L-cysteine were obtained in the same manner. The involvement of CaSRs in L-cysteine-induced relaxations was investigated in the presence of Calhex-231, a CaSRs inhibitor. After the relaxant responses to L-cysteine (1 μM-10mM) were obtained, tissues were washed and incubated with 5 μM Calhex-231 for 30 min, and then responses to L-cysteine (1 μM-10 mM) were obtained in the same manner.

### Measurement of endogenous H_2_S release in mouse bladder strips

H_2_S levels were measured as described in our previous studies [29,36]. In the presence of Fe^3+^, H_2_S reacts with color developing agent to form stable methylene blue, and methylene blue has the maximum absorption peak at 665 nm. The H_2_S content can be calculated by measuring its absorption value. H_2_S production in bladder tissue samples was determined with a commercially available H_2_S colorimetric assay kit (Elabscience Biotechnology Co., Ltd., Wuhan, China) through the reaction between H_2_S and zinc acetate, N, N-dimethyl-p-phenylenediamine, and ammonium ferric sulfate. Protein concentration was determined by using a bicinchoninic acid assay kit (Sigma Chemical Co., St. Louis, MO, USA). Bladder tissues at 10 % (w/v) concentration were homogenized in normal saline (0.9 %) at 4 °C. And then centrifuged for 10 min at 4 °C at 10000× g to remove insoluble material, and the supernatant was collected. The supernatant solution was mixed with an equal volume of reagents 1 and 2. After centrifugation, the sediment was dissolved in reagents 1, 3, and 4. The supernatant obtained after centrifugation was mixed with reagent 5. The absorbance of solutions was measured after 20 min at a wavelength of 665 nm and H_2_S concentrations in bladder tissues, expressed as nmol/mg protein.

### Drugs

The following drugs were used; amino-oxyacetic acid (o-carboxymethyl), dl-propargylglycine, carbachol, L-cysteine (Sigma Chemical Co., St Louis, MO, USA), Calhex-231 (4-Chloro-N-[(1S,2S)-2-[[(1R)-1-(1-naphthalenyl)ethyl]amino]cyclohexyl]-benzamide hydrochloride) (CAYMAN Chemical Company, USA) and; CaCl_2_ (MERCK). All drugs were dissolved in distilled water except calhex-321, which was dissolved in dimethyl sulphoxide (DMSO) up to 1 mM; further dilutions were made in distilled water. DMSO per se did not affect the tone of the strips. The final concentration of DMSO was less than 0.001 M.

### Statistical analysis

The relaxant responses to CaCl_2_ and L-cysteine were expressed as a percentage of the carbachol-induced contraction. Due to CaCl_2_ was applied to the tissues as a single series, the single-series relaxations of the inhibitor groups were compared with those in the control group. Also, since a difference was observed between the first and second relaxation series in the L-cysteine control group, the relaxant responses to L-cysteine obtained in the presence of specific inhibitors were compared to the second series of the control group. Student unpaired *t*-tests and analysis of variance (ANOVA) were used for statistical comparison of mean values, and corrected for multiple comparisons (Bonferroni corrections). All data are presented as means ± standard error of the mean (SEM), and “n” refers to the number of tissues used in each experiment. Maximum relaxant response (Emax) was expressed as the relaxation induced by CaCl_2_ and L-cysteine. The sensitivities of the bladder tissues to CaCl_2_ and L-cysteine were calculated as the effective concentration the elicits 50 % of the maximal response by using nonlinear regression curve fit and expressed as pEC50 (-Log M) (GraphPAD Software, version 5.00, San Diego, USA). P<0.05 was considered to be statistically significant.

## Results

### The role of L-cysteine/H_2_S pathway on CaCl_2_-induced relaxations

**Fig. 1:**
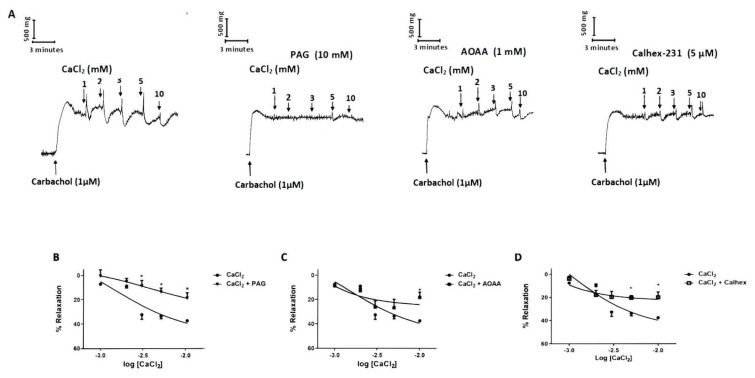
The role of L-cysteine/H_2_S pathway on CaCl_2_-induced relaxations. Representative traces CaCl_2_-induced relaxations (**A**). Graph showing that CaCl_2_-induced relaxations in the presence of PAG (Cystathionine-gamma-lyase (CSE) inhibitor, 10 mM) (**B**), AOAA (Cystathionine beta synthase (CBS) inhibitor, 1 mM) (**C**), and Calhex-231 (Calcium Sensing Receptors (CaSRs) inhibitor, 5 μM) (**D**). All values are mean ± S.E.M. (n=6). * P<0.05 significantly different from the control; unpaired *t*-test followed by Bonferroni’s comparison test.

To elucidate the relaxant effect of CaCl_2_ on carbachol-induced contractions in mouse bladder strips, the effects of CaCl_2_ were studied. After a steady-state contraction was obtained with carbachol (1 μM), CaCl_2_ was applied at 1, 2, 3, 5 and 10 mM concentrations to the bladder tissues. CaCl_2_ caused concentration-dependent relaxations in bladder tissues pre-contracted with carbachol (1 μM) (Fig. 1A). To determine the involvement of L-cysteine/H_2_S pathway in the relaxant action of CaCl_2_ in mouse bladder tissues, we investigated the inhibitory effects of PAG (10 mM) and AOAA (1 mM), CSE and CBS inhibitor, respectively, on relaxations induced by CaCl_2_ (1, 2, 3, 5 and 10 mM). Pre-incubation of bladder strips with PAG and AOAA significantly reduced the relaxant responses to CaCl_2_ (P<0.05; Fig. 1A, B, C). Emax to CaCl_2_ were significantly decreased by PAG and AOAA from 37.40±1.30 % to 18.87±4.37 % and 18.20±4.34 %, respectively (P<0.05). But there was no significant difference in pEC50 values for CaCl_2_ between control (2.89±0.39), PAG (2.25±0.40) and AOAA (2.72±0.37) groups.

To confirm that the relaxant effect of CaCl_2_ is mediated through CaSR, we examined the effect of Calhex-231, a CaSRs inhibitor, on CaCl_2_-induced relaxations in the bladder tissues. Pre-incubation of bladder tissues with Calhex-231 (5 μM) significantly reduced the relaxant responses to CaCl_2_ (P<0.05; Fig. 1A and 1D). Emax to CaCl_2_ were significantly decreased by Calhex-231 from 37.40±1.30 % to 19.75±4.57 % (P<0.05). However, there was no significant difference in the pEC50 values for CaCl_2_ between control (2.89±0.39) and Calhex-231 (2.61±0.37) groups.

### The role of CaSR on L-cysteine-induced relaxations

**Fig. 2:**
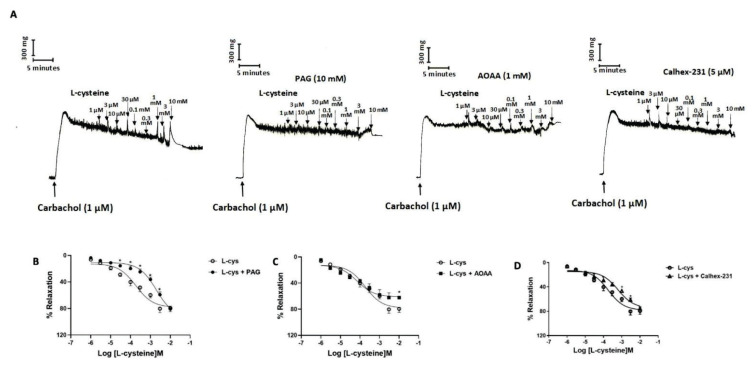
The role of Calcium Sensing Receptors (CaSRs) on L-cysteine-induced relaxations. Representative traces L-cysteine-induced relaxations (**A**). Graph showing that L-cysteine-induced relaxations in the presence of PAG (Cystathionine-gamma-lyase (CSE) inhibitor, 10 mM) (**B**), AOAA (Cystathionine beta synthase (CBS) inhibitor, 1 mM) (**C**) and Calhex-231 (CaSR inhibitor, 5 μM) (**D**) in mouse bladder strips All values are mean ± S.E.M. (n=6). * P<0.05 significantly different from the control; unpaired *t*-test followed by Bonferroni’s comparison test.

To investigate the involvement of CaSR in relaxant responses to endogenous H_2_S substrate L-cysteine in bladder strips, we studied the inhibitory effects of Calhex-231, a CaSRs-specific inhibitor, on relaxations induced by L-cysteine. After a steady-state of contraction was obtained with carbachol (1 μM), L-cysteine was applied cumulatively at concentrations from 1 μM to 10 mM. L-cysteine (1 μM-10 mM) caused concentration-dependent relaxations in bladder strips pre-contracted with carbachol (1 μM) (Fig. 2A).

To confirm that endogenous H_2_S-dependent relaxation of L-cysteine, we studied the effect of H_2_S synthesis inhibitors on L-cysteine-induce relaxations. PAG (10 mM), a CSE inhibitor and AOAA (1 mM), a CBS inhibitor, significantly decreased the relaxant responses to L-cysteine (1 μM-10 mM) (P<0.05; Fig. 2A, B and C). Also, pEC50 for L-cysteine was significantly reduced by PAG from 3.88±0.15 to 2.99±0.15 (P<0.05). The Emax for L-cysteine were significantly decreased by AOAA 79.27±5.47 % to 62.06±2.43 % (P<0.05). However, there was no significant difference in pEC50 values for L-cysteine between the control (3.88±0.15) and AOAA (3.79±0.15) groups.

To determine the involvement of CaSRs in the relaxant action of L-cysteine/H_2_S in mouse bladder tissues, we investigated the inhibitory effects of Calhex-231 on relaxations induced by L-cysteine (1 μM-10 mM). Pre-incubation of bladder strips with Calhex-231 significantly reduced the relaxations at lower concentrations of L-cysteine (P<0.05; Fig. 2A and 2D) but the Emax value did not change. The pEC50 for L-cysteine was significantly reduced by Calhex-231 from 3.88±0.15 to 3.31±0.15 (P<0.05).

### Effects of CaCl_2_ and Calhex-231 on H_2_S generation in mouse bladder tissue

**Fig. 3:**
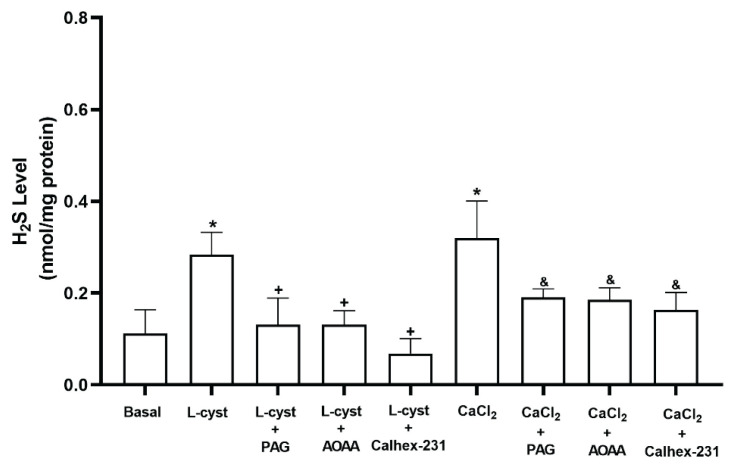
The role of CBS, CSE and Calcium Sensing Receptors (CaSRs) inhibition on endogenous H_2_S formation. The bar graph shows the effects of L-cysteine (L-cyst; 10 mM), and CaCl_2_ (10 mM) in the absence or presence of PAG (Cysta-thionine-gamma-lyase (CSE) inhibitor, 10 mM), AOAA (Cystathionine beta synthase (CBS) inhibitor, 1 mM), and Calhex-231 (CaSRs inhibitor, 5 μM), All values are mean ± S.E.M. (n=4). * P<0.05 significantly different from basal; ^+^ P<0.05 significantly different from L-cysteine; ^&^ P<0.05 significantly different from CaCl_2_; unpaired *t*-test followed by Bonferroni’s comparison test.

We studied the effects of CaCl_2_ and Calhex-231 on H_2_S generation. Mouse bladder tissue generated detectable amounts of basal H_2_S (0.11±0.02 nmol/mg). L-cysteine increased basal H_2_S generation (0.28±0.05 nmol/mg), and CSE inhibitor PAG (10 mM) and CBS inhibitor AOAA (1 mM) reduced the increase in H_2_S production induced by L-cysteine from 0.28±0.05 nmol/mg to 0.13±0.03 nmol/mg and 0.13±0.02 nmol/mg respectively, suggesting that mouse bladder tissue is capable of synthesizing H_2_S from L-cysteine. Also, Calhex-231, a CaSRs-specific inhibitor, reduced the increase in H_2_S production in the presence of L-cysteine (0.07±0.02 nmol/mg). In addition, CaCl_2_ increased basal H_2_S generation (0.31±0.04 nmol/mg), and PAG, AOAA and Calhex-231 reduced the increase in H_2_S production induced by CaCl_2_ from 0.32±0.04 nmol/mg to 0.19±0.01 nmol/mg, 0.18±0.01 nmol/mg and 0.16±0.02 nmol/mg respectively, (Fig. 3), suggesting an interaction between H_2_S and CaSRs pathway, and the interaction may be occur through the CSE and CBS enzyme in the mouse bladder tissue.

## Discussion

In the present study, we investigated the role of L-cysteine/H_2_S pathway in CaSR-mediated responses in mouse bladder tissue. We found that 1) CaCl_2_ produced relaxant responses. 2) CaCl_2_-induced relaxations were inhibited by Calhex-231, a CaSR-specific inhibitor. 3) L-cysteine-induced-relaxations were inhibited by PAG and AOAA, CSE and CBS enzyme inhibitors, respectively. 4) PAG and AOAA reduced the relaxations to CaCl_2_. 5) Calhex-231 reduced the relaxations to L-cysteine. 6) Calhex-231 reduced the increase in H_2_S production in the presence of L-cysteine. In addition, CaCl_2_ increased basal H_2_S generation, and PAG, AOAA and Calhex-231 reduced the increase in H_2_S production stimulated with CaCl_2_. These findings suggest that there is an interaction between L-cysteine/H_2_S pathway and CaSRs, and CSE/CBS-induced endogenous H_2_S may partly contribute to the relaxation responses due to CaSRs activation in mouse bladder.

CaSRs, a G-protein coupled receptor, trigger intracellular signals *via* the modulation of a series of intracellular signaling proteins and modulate several physiological functions. The presence of CaSRs has been demonstrated in aortic endothelial cells and vascular smooth muscle cells including human artery [7,8,37,38]. Also, it has been reported that CaSRs are expressed in rat bladder urothelium and activation of these receptors reduces detrusor activity [28]. H_2_S is a gaseous neurotransmitter that has a relaxing effect on vascular and extravascular smooth muscles. H_2_S is synthesized endogenously from L-cysteine *via* CSE, CBS and 3-MST enzymes in mammalian tissues. It has been reported that H_2_S is synthesized in mouse, rat, pig and human bladder tissues [25,27,29,39]. H_2_S produced a concentration-dependent contraction and relaxation response in isolated bladder tissues [25,40]. In the present study, we obtained concentration-dependent relaxation to L-cysteine in mouse bladder. Taken together, we propose that the effect of L-cysteine on bladder smooth muscle tone may vary depending on the type of pre-treatment and its concentration. It has been reported that H_2_S synthesis is associated with an increase in the amount of intracellular Ca^2+^in vascular smooth muscle cells and CaSRs activation increases CSE expression and H_2_S synthesis in vascular smooth muscle [33]. However, it has not been previously investigated the interaction between H_2_S and CaSRs in bladder smooth muscle tissue. In the present study, we investigated the role of L-cysteine/H_2_S pathway in CaSRs-mediated responses in mouse bladder tissue. For this purpose, CaCl_2_ was applied as a bolus into bladder tissues contracted by carbachol for CaSRs activation, and CaCl_2_ produced concentration-dependent relaxations. To confirm that CaCl_2_ relaxation is mediated *via* CaSRs, CaCl_2_-induced relaxant responses were studied in the presence of Calhex-231, negative allosteric modulators of CaSRs or calcilytics [15,41,42]. CaCl_2_ relaxations significantly decreased in the presence of Calhex-231, suggesting that CaCl_2_ responses are dependent on CaSRs activation in bladder tissue. In consistent with our findings, it has been reported that CaCl_2_-induced relaxant responses were significantly reduced in the presence of Calhex-231 in rat and rabbit mesenteric artery tissues, suggesting that CaSRs mediate CaCl_2_ relaxations [8,14,17]. In our study, we aimed to investigate the role of endogenous H_2_S in CaSRs-mediated relaxations, we studied the effects of PAG and AOAA, CSE and CBS enzyme inhibitor, respectively, on CaCl_2_-induced relaxant responses. PAG and AOAA caused a significant decrease in CaCl_2_ relaxation responses. This finding functionally suggests that CaCl_2_-mediated relaxations may partly dependent on CSE/CBS-induced endogenous H_2_S formation. Consistent with this, we observed that CaCl_2_ enhanced basal H_2_S formation, and PAG, AOAA and Calhex-231 markedly reduced the augmentation in H_2_S production in the presence of CaCl_2_, indicating that the interaction between CaSRs and L-cysteine/H_2_S pathway mainly occurs through the CSE and CBS enzymes in the mouse bladder tissues. Also, it has been reported that CSE-induced H_2_S synthesis increases by CaSRs activation with CaCl_2_ in VSMCs [33].

Furthermore, there may be a two-way interaction between CaSRs and H_2_S, such as CaCl_2_ increasing H_2_S production through CSE/CBS enzyme activation and H_2_S causing relaxation by activating CaSRs. Consequently, we studied the role of CaSRs in relaxant responses to L-cysteine/H_2_S in bladder tissues. L-cysteine, H_2_S substrate, caused a concentration-dependent relaxation response on carbachol-constricted isolated mouse bladder tissues. In the present study, inhibition of L-cysteine-induced relaxation responses in the presence of PAG and AOAA confirms that these relaxations are caused by endogenous H_2_S. Also, these findings functionally demonstrate the role of CSE and CBS enzymes in endogenous H_2_S synthesis in mouse bladder tissue. Similar to our findings, Fusco *et al*. showed that L-cysteine-induced relaxations were significantly reduced in the presence of PAG and AOAA in isolated human bladder tissue [27]. Also, expressions of CSE, CBS, and 3-MST enzymes and L-cysteine-mediated H_2_S production were shown in mouse, rat, guinea pig, and human bladder tissues [24–26,39,43]. Consistent with studies, we recently reported the presence of endogenous H_2_S-generating enzymes in mouse bladder tissue [29]. To determine the possible contribution of CaSRs to endogenous H_2_S relaxation responses, the effect of Calhex-231 on L-cysteine-induced relaxant responses were investigated. In the presence of Calhex-231, a significant decrease in L-cysteine-induced relaxations was observed, and Calhex-231 reduced the increase in H_2_S production in the presence of L-cysteine, suggesting that endogenous H_2_S responses may be associated with CaSRs in mouse bladder tissue. To our knowledge, this is the first report to show the involvement of CaSRs on endogenous L-cysteine/H_2_S pathway in bladder tissues. Some studies also confirmed that CaSRs activation mediated the H_2_S enzymes pathway in vascular smooth muscle cells [33,34]. Also, Zhong *et al*. demonstrated that CaSRs regulated the endogenous CSE/H_2_S pathway to inhibit the proliferation of vascular smooth muscle cells in both diabetic and high glucose models [33]. In addition, it has been reported that CaSR modulates CSE/H_2_S pathway and is associated with PLC-IP3 receptor and calmodulin signaling which inhibits the proliferation of VSMCs *via* the Erk1/2 dependent signaling pathway in hyperhomo-cysteinemia [34]. Our results are in agreement with previous studies showing the contribution of CaSRs to endogenous H_2_S-induced physiological responses. Further studies are needed to clarify the interaction between L-cysteine/H_2_S and CaSRs pathway in bladder tissue.

In conclusion, these results suggest that there is an interaction between L-cysteine/H_2_S and CaSRs through activation of CBS and CSE enzymes. Also, the mechanism of CaSRs-mediated relaxant responses involves, at least in part, CSE and CBS-generated H_2_S in mouse bladder. There may be a two-way interaction between CaSRs and H_2_S, such as CaCl_2_ increasing H_2_S production through CSE/CBS enzyme activation and H_2_S causing relaxation *via* activation of CaSRs. This is the first time the interaction between CaSRs and L-cysteine/H_2_S pathway has been demonstrated in bladder tissue.
